# Heterogeneities in dengue spatial-temporal transmission in Brazilian cities and its influence on the optimal age of vaccination

**DOI:** 10.1186/s12889-019-6426-9

**Published:** 2019-02-06

**Authors:** Luciana L. Cardim, Suani T. R. Pinho, M. Gloria Teixeira, M. Conceição N. Costa, M. Lourdes Esteva, Claudia P. Ferreira

**Affiliations:** 1 Instituto de Saúde Coletiva, Universidade Federal da Bahia, Salvador, 40.110-140 Brazil; 2 Instituto de Física, Universidade Federal da Bahia, Rua Caetano Moura, Campus Universitário de Ondina, Salvador, 40.210-340 Brazil; 3 Facultad de Ciencias, Universidad Nacional Autónoma de México, México, 04510 México; 4 São Paulo State University (UNESP), Institute of Biosciences, Department of Biostatistics, Botucatu, 18618-000 Brazil

**Keywords:** Mathematical model, Age profile, Vaccination strategies

## Abstract

**Background:**

The development of a safe and effective vaccine is considered crucial for dengue transmission control since vetor control has been failed; some potential candidates are currently in test, and in this context theoretical studies are necessary to evaluate vaccination strategies such as the age groups that should be vaccinated, the percentage of the population at risk, and the target geographic regions to make dengue control feasible and optimal.

**Methods:**

A partial differential model is used to mimics dengue transmission in human population in order to estimate the optimal vaccination age, using data collected from dengue reported cases in ten cities of Brazil from 2001 to 2014. For this purpose, the basic reproduction number of the disease was minimized assuming a single-dose vaccination strategy, equal vaccine efficacy for all circulating serotypes, and no vaccine failure. Numerical methods were used to assess the optimal vaccination age and its confidence age range.

**Results:**

The results reveal complex spatial-temporal patterns associated to the disease transmission, highlighting the heterogeneity in defining the target population for dengue vaccination. However, the values obtained for the optimal age of vaccination, as targeting individuals under 13 years old, are compatible with the ones reported in similar studies in Brazil. The results also show that the optimal age for vaccination in general does not match with the age of the highest number of cases.

**Conclusions:**

The variation of the optimal age for vaccination across the country reflects heterogeneities in dengue spatial-temporal transmission in Brazilian cities, and can be used to define the target population and cities to optimize vaccination strategies in a context of high cost and low quantity of available vaccine.

## Background

Control methods of infectious diseases must take into account ecological, immunological, and behavioral aspects of the disease agent, as well as its transmission mode and the types of hosts. For this end, analysis of spatial-temporal patterns of infectious diseases reports, together with laboratory and field experiments, are important tools for understanding diseases transmission, and designing control strategies, such as sanitary measures, health policies, vaccination, or vector control in the case of vector-borne diseases.

One of the most efficient control tools has been the vaccine. Several diseases that plagued humanity for centuries, such as poliomyelitis and measles, have been currently controlled in some regions or countries, thanks to vaccination routine or campaigns. The most notorious example is smallpox, which was eradicated in 1979, by a collaborative global vaccination program [1].

For vector-borne diseases, the control is based mainly on the reduction of the vector population. In particular, for dengue, all efforts have been focused on *Aedes* mosquitoes control, by means of the use of insecticides that eliminate adult stage, larvicides and destruction of oviposition containers to remove immature stage, and by the use of biological techniques [2–4]. Unfortunately, increasing urbanization, waste generation, human displacement, mosquito resistance to insecticides, as well as mosquito fast spread across world, have contributed to vector control failure [2].

The development of safe and effective vaccines is considered crucial for better expectations for dengue control. The principal obstacle is the existence of four different serotypes, and the potential risk of dengue hemorrhagic fever (DHF) associated to secondary infections with heterologous serotype [5]. Due to these dengue-specific complexities, vaccine development focuses on the generation of a tetravalent vaccine aimed to provide long-term protection against all virus serotypes. Some vaccine candidates are currently in test, and only one was registered until now in some countries [6]. It is worth to mention that, at the end of 2017, there was an increment in the number of hospitalizations for severe dengue in vaccinated individuals who had never had dengue before [7].

Theoretical studies, such as mathematical modeling, are useful to provide tools for the development of vaccination strategies that aim to promote herd immunity. Using dengue data, these models can be useful to decide which are the age groups that should be vaccinated, the percentage of the population at risk that must be vaccinated to control disease transmission, and the target geographic regions to optimize disease control.

Mathematical models have been proposed to evaluate possible dengue vaccination strategies using different techniques. Billings et al. (2008) [8] used a system of ordinary differential equations (ODE) to evaluate the efficacy of a single-strain vaccine assuming antibody-dependent enhancement. The authors considered several possible scenarios to evaluate the vaccine efficacy in the presence of two serotypes, resulting in a diagram showing effective vaccination rates versus strains persistence and extinction. Amaku et al. (2012) [9] used dengue serological data from Recife City, Brazil, and a system of time-delayed differential equations to estimate the optimal vaccination age; they found that it should be vaccinated children between 3 and 14 years, and 80% of vaccination coverage has to be achieved. On the other hand, an agent-based model was developed by Chao et al. [10] to simulate the epidemiology of dengue transmission in a semi-rural area of Thailand; they obtained that, for a fixed number of doses, vaccinating children from 2 to 14 years old would reduce dengue infection in the total population more than covering both children and adults (2 to 46 years old).

An age-structured multi-strain model was done by the authors of reference [11] to design scenarios for the potential impact of a dengue vaccine on a population. Using data from Southern Vietnam, the authors showed that seasonality and short cross-protection against infection ranging from 6 to 17 months are a keystone to produce the observed disease periodicity. Also they argued that vaccination reduces disease burden by reducing the magnitude and frequency of outbreaks. The same kind of model was used to set up the vaccination strategy that minimizes the incidence of DHF [12]: in Thailand the optimal strategy is to vaccine children from 0.5 to 12 years old while in Brazil, it is better to vaccine adults from 18 to 34 years old. Recently, using a system of partial differential equations for human population, and delay differential equations for vector population, as well as dengue incidence data in Brazil, Maier et al. [13] provided an estimation of the optimal vaccination age when different assumptions for vaccine efficacy and risk of infection have been taking into account; minimizing hospitalization or mortality due to dengue as measures of risk of dengue infection, wide ranges of values of optimal vaccination ages have been obtained, regarding both the serotypes in circulation and the model assumptions.

The objective of this study is to estimate the priority vaccination age range against the four serotypes of dengue for several Brazilian cities that had heterogeneities in spatial-temporal dengue transmission for the period of 2001 to 2014. For this end, we use the partial differential equations (PDE) model proposed by Cruz-Pacheco et al. [14]. The variation of the optimal age for vaccination across the country can be used to define the target population, taking into account dengue epidemics in that period in different cities, to optimize vaccination strategies in a context of high cost and low quantity of available vaccine.

## Methods

The method we used to evaluate the optimal vaccination age [14] in ten selected Brazilians cities, as well as in the whole country, is based on continuous compartamental models. These models are applied for large enough populations, since they are based on the assumption that susceptible and infectious are well mixed. Also vector-borne diseases have a higher prevalence in regions with high density of human and vectors. For these reasons, we establish the following criteria to select the cities used in the present study: (i) the city has to have more than 500,000 inhabitants; (ii) it has to present a cumulative incidence of dengue fever during the period of 2001 to 2014 greater than 500 per 100,000 inhabitants.

The number of dengue cases by year (2001 to 2014) and by age (< 1 year, 1 to 4 years, 5 year interval from 5 to 59 years, > 60 years) were obtained from Brazilian Notified Disease Information System (SINAN). The midpoints of these age classes were used in the analyses.

In the epidemiological model, the human population is divided into Susceptible, Infected and Recovered (SIR model), and the vector population is divided into Susceptible and Infected (SI model). The coupling between the two population is done by the transmission rate between infected humans (or susceptible vectors) and susceptible vectors (or infected humans).

Supposing that the increase of human protection under an age-vaccination distribution is equivalent to the decrease of the net reproductive number *R*_0_(*V*) constrained by the cost of vaccination, Cruz-Pacheco et al. [14] showed that minimizing *R*_0_(*V*) is equivalent to maximize the function *H*(*a*) given by:

1 $$ H(a) = { K e^{\mu_{h} a}\int_{a}^{\infty}f(a^{\prime})e^{-\mu_{h} a^{\prime}}da^{\prime}},  $$

where *f*(*a*) is the probability that a susceptible human of age *a* acquires dengue virus from an infected vector and it is obtained from the distribution of dengue cases by age. In the above expression, *K* is given by

2 $$ K= { b \alpha_{v} N_{v} \mu_{h} \over c N_{h} \mu_{v} \left(\mu_{h} + \gamma_{h}\right)},  $$

where *b* is the mosquito biting rate, *μ*_*v*_ is the vector mortality rate, *α*_*v*_ is the infection rate from vectors to humans, *μ*_*h*_ is the human mortality rate, *γ*_*h*_ is the human recovery rate, and *c* is the cost associated to the vaccination schedule. Finally, *N*_*v*_ and *N*_*h*_ are, respectively, the total number of vectors and humans whose values depend on each city. As in reference [14], we assume that *b*=0.5 days ^−1^, *α*_*v*_=0.75, *μ*_*h*_=0.00004 days ^−1^, *c*=1, *N*_*v*_/*N*_*h*_=1, *μ*_*v*_=0.06 days ^−1^, and *γ*_*h*_=0.125 days ^−1^.

The optimal vaccination age corresponds to the age for which *H*(*a*) assumes its maximum value. All parameters in the expression for K are constant (do not depend on age *a*), therefore their values are irrelevant for the evaluation of the optimal vaccination age. The exception is the *μ*_*h*_ parameter that also appears inside of the integral multiplying *a*; the optimal vaccination age can be sensitive to it. We are assuming that everybody is vaccinated at the same age, vaccine efficacy is constant along the age classes, and the same for all serotypes; also that vaccine-induced immunity is lifelong.

Using data obtained from SINAN [15], the optimal vaccination age for each city in each year was obtained by the following algorithm: (i) evaluation of *f*(*a*) from the proportion of dengue reported cases; (ii) approximation of the integral that appears in equation (1) by the trapezoidal rule resulting in a curve of *H*(*a*) that is a function of *a*; (iii) evaluation of the derivative of this curve by the method of finite differences to obtain another curve that is also a function of *a*, *h*(*a*):=*dH*(*a*)/*da*; iv) the use of Newton’s method to find the roots of *h*(*a*). As we have a discrete set of data instead of a curve, we used two different approaches: 1) a linear interpolation in the interval of *h*(*a*_1_) and *h*(*a*_2_) where *h*(*a*_1_)*h*(*a*_2_)<0, to estimate ${\bar a}\in ]a_{1},a_{2}[$, the optimal vaccination age; 2) a cubic spline to interpolate all the curve before searching for *h*(*a*_1_)*h*(*a*_2_)<0. The results obtained from these two approaches were compared to define the confidence interval of the optimal vaccination range. We decided to report the results obtained from the linear interpolation, except in cases where the linear interpolation failed on getting the optimum value of ${\bar a}$.

In Brazil, the serology for dengue is done in a small fraction of the reported cases during the epidemic and non-epidemic period, when dengue incidence is below than 300 per 100.000 habitants [16]. After that, all diagnoses are clinical, based on the individual symptoms, and area of circulation of the dengue virus. Overall, it is estimated from the literature that the percentage of dengue asymptomatic individuals can reach up to 80% of the cases [17]. Therefore, we assumed that the age distribution of dengue cases is not perfect, and we reported the optimal vaccination age as well as its confidence age range. In order to construct a confidence interval for the optimal vaccination age, we generated 100,000 samples obtained from the dengue reported cases, assuming an error of 15% in each one of them. This value of 15% was chosen after a systematic study that involved the error coming from the use of numerical methods (derivation, integration, root finding, and interpolation) to find the optimal vaccination age. Besides the uncertainty on the reported cases of dengue, variation on the human mortality rate can affect the optimal vaccination age (see Eqs. (1) and (2)). In order to investigate that effect, the same procedure was applied to estimate the optimal vaccination age, as well as its confidence age range, assuming different values for human mortality rate. Since from 2001 to 2014, the life expectancy in Brazil changes from 70.5 to 75 years [18], we used the minimal (Table 1) and the maximal (Table 2) values of life expectancy in the estimations for each city at each year.

**Table 1:** Optimal age and confidence interval for dengue vaccination per year from 2001 to 2014 for the whole country, Brazil (Br) and for the analysed Brazilian cities

City	2001	2002	2003	2004	2005	2006	2007	2008	2009	2010	2011	2012	2013	2014
Br.	**8.6**	**7.5**	**8.3**	**8.4**	**7.6**	**7.9**	**7.1**	**3.2**	**4.5**	**6.6**	**4.6**	**6.8**	**8.6**	**8.4**
	[7.2,10.3]	[6.0,9.2]	[7.0,9.9]	[7.2,10.0]	[6.2,9.1]	[6.4,9.5]	[5.5,8.7]	[2.3,8.5]	[3.2,6.0]	[5.3,8.1]	[3.5,6.0]	[5.5,8.1]	[7.4,10.1]	[7.0,10.1]
Fo	**7.6**	**8.5**	**6.1**	**8.2**	**3.4**	**4.1**	**NA**	**NA**	**2.5** ^∗^	**3.0** ^∗^	**3.4** ^∗^	**6.2**	**5.8**	**5.1**
	[5.8,9.5]	[7.2,10.0]	[4.8,7.8]	[7.0,9.8]	[2.4,9.2]	[3.1,5.3]	[0.0,5.7] ^∗^	[0.0,5.6] ^∗^	[0,5.6] ^∗^	[0,4.7] ^∗^	[0.0,4.6] ^∗^	[5.0,7.6]	[4.7,7.3]	[3.8,7.0]
Na	**10.4**	**9.9**	**11.1**	**8.7**	**8.2**	**5.1**	**4.0**	**3.4** ^∗^	**4.0**	**6.1**	**3.9**	**6.6**	**11.6**	**5.7**
	[8.8,12.4]	[8.3,12.0]	[9.4,12.8]	[7.5,10.3]	[6.7,9.8]	[3.4,7.6]	[2.5,6.1]	[2.8,5.9] ^∗^	[1.5,10.1]	[4.8,7.6]	[2.7,5.1]	[5.3,8.3]	[8.6,13.4]	[2.4,13.0]
Ma	**10.5**	**12.1**	**10.8**	**8.2**	**8.6**	**7.9**	**6.1**	**3.3**	**4.5**	**2.5**	**3.3**	**7.7**	**3.3** ^∗^	**8.0**
	[8.3,15.0]	[10.6,13.7]	[9.6,12.3]	[7.3,9.4]	[7.6,9.9]	[6.9,9.0]	[4.7,7.6]	[2.3,7.5]	[3.3,5.9]	[2.1,5.9]	[2.1,5.5]	[6.5,9.0]	[2.8,9.6] ^∗^	[6.7,9.3]
BH	**10.6**	**10.0**	**9.8**	**11.0**	**8.6**	**10.1**	**9.5**	8.1	**8.1**	**8.5**	**9.0**	**10.6**	**9.0**	**8.3**
	[9.5,12.2]	[8.9,11.5]	[8.7,11.1]	[9.9,12.4]	[7.7,9.7]	[8.9,11.7]	[8.5,10.8]	[7.0,9.4]	[6.9,9.3]	[7.4,9.9]	[7.9,10.4]	[9.1,12.8]	[8.0,10.3]	[7.0,10.0]
RJ	**9.4**	**7.6**	**6.9**	**9.2**	**5.4**	**8.3**	**6.2**	**2.6**	**4.9**	**3.3**	**2.8**	**6.6**	**8.1**	**2.7** ^∗^
	[8.1,10.9]	[6.1,9.4]	[5.3,8.6]	[7.8,11.0]	[3.3,7.9]	[6.9,9.8]	[5.2,7.5]	[2.1,7.5]	[3.2,7.0]	[2.4,6.5]	[2.3,5.3]	[5.4,7.8]	[6.7,9.5]	[2.2,10.8] ^∗^
Ca	**7.8**	**7.2**	**10.0**	**19.0** ^∗^	**2.3** ^∗^	**5.1**	**8.7**	**8.1**	**3.2**	**10.1**	**8.5**	**9.7**	**8.8**	**9.4**
	[3.1,10.5]	[5.2,9.1]	[7.9,12.4]	[17.4,20.5] ^∗^	[1.8,20.3] ^∗^	[1.1,8.6]	[7.3,10.3]	[4.2,12.5]	[2.1,12.4]	[8.6,12.2]	[7.2,10.2]	[8.4,11.7]	[7.7,10.2]	[8.2,11.0]
RP	**11.3**	**11.8**	**10.6**	**9.1**	**12.4**	**9.6**	**10.4**	**9.1**	**8.1**	**6.6**	**6.3**	**8.1**	**7.7**	**9.0**
	[10.0,13.0]	[10.5,13.1]	[9.4,12.2]	[6.5,10.2]	[11.1,14.0]	[8.4,11.2]	[8.9,12.3]	[8.0,10.6]	[6.9,9.4]	[5.4,8.1]	[5.1,7.7]	[7.0,9.4]	[6.4,9.1]	[7.6,10.8]
CG	**8.4**	**8.0**	**9.7**	**13.2**	**12.9** ^∗^	**8.1**	**8.1**	**8.5**	**6.8**	**6.2**	**3.3** ^∗^	**8.8**	**8.7**	**5.2**
	[7.1,9.8]	[6.8,9.3]	[8.6,11.0]	[0.0,17.0]	[12.0,14.0] ^∗^	[6.9,9.5]	[6.8,9.5]	[6.5,10.7]	[5.2,8.7]	[5.0,7.7]	[2.7,7.5] ^∗^	[7.6,10.4]	[7.5,10.2]	[3.1,7.5]
Cu	**10.3**	**5.6**	**5.1**	**13.5**	**9.9**	**6.4**	**3.5**	**2.7** ^∗^	**2.4** ^∗^	**2.8** ^∗^	**2.3** ^∗^	**5.9**	**6.1**	**2.8**
	[9.1,11.8]	[4.2,7.4]	[4.1,6.4]	[0.0,15.1]	[7.8,14.0]	[5.4,7.7]	[2.5,6.7]	[2.3, 5.7] ^∗^	[2.1,5.5] ^∗^	[2.1,4.9] ^∗^	[2.0,3.8] ^∗^	[4.8,7.2]	[4.6,7.8]	[2.2,7.6]
Go	**9.4**	**6.9**	**8.8**	**9.5**	**8.4**	**7.3**	**8.5**	**7.6**	**8.5**	**8.3**	**7.2**	**8.4**	**9.2**	**9.4**
	[8.3,10.7]	[5.1,8.3]	[7.7,10.1]	[8.4,11.0]	[7.1,10.0]	[5.9,9.0]	[7.3,9.9]	[6.2,9.3]	[7.1,10.0]	[6.8,9.9]	[5.5,9.1]	[6.8,10.3]	[7.8,10.9]	[8.2,10.9]

The letters Fo, Na, Ma, BH, RJ, Ca, RP, CG, Cu and Go are the abbreviations for Fortaleza, Natal, Maceió, Belo Horizonte, Rio de Janeiro, Campinas, Ribeirão Preto, Campo Grande, Cuiabá, Goiânia, respectively. Non Applied (NA) means that neither the maximum value of age nor the confidence interval was found. The symbol ^∗^ highlights the values obtained after a cubic spline interpolation was done in the data. For the others values, a linear interpolation was used. Human mortality rate *μ*_*h*_=1./70.5 years ^−1^

**Table 2:** Optimal age and confidence interval for dengue vaccination per year from 2001 to 2014 for the whole country, Brazil (Br) and for the analysed Brazilian cities

City	2001	2002	2003	2004	2005	2006	2007	2008	2009	2010	2011	2012	2013	2014
Br.	**8.3**	**7.2**	**8.1**	**8.1**	**7.3**	**7.6**	**6.7**	**3.0**	**4.2**	**6.3**	**4.4**	**6.5**	**8.3**	**8.1**
	[6.9,9.9]	[5.7,8.8]	[6.7,9.5]	[6.9,9.6]	[5.9,8.7]	[6.1,9.2]	[5.2,8.3]	[2.3,8.0]	[2.9,5.7]	[5.0,7.8]	[3.2,5.7]	[5.2,7.8]	[7.1,9.7]	[6.6,9.7]
Fo	**7.2**	**8.2**	**5.8**	**8.0**	**3.2**	**3.8**	**NA**	**NA**	**2.4** ^∗^	**2.8** ^∗^	**3.3** ^∗^	**5.9**	**5.5**	**4.8**
	[5.5,9.0]	[6.9,9.6]	[4.6,7.4]	[6.6,9.4]	[2.3,9.2]	[2.9,5.0]	[0.0,5.4] ^∗^	[0.0,5.3] ^∗^	[0,4.8] ^∗^	[0,4.5] ^∗^	[0.0,4.4] ^∗^	[4.7,7.3]	[4.4,7.0]	[3.5,6.5]
Na	**10.0**	**9.6**	**10.7**	**8.4**	**7.9**	**4.7**	**3.7**	**3.3** ^∗^	**NA**	**5.8**	**3.6**	**6.3**	**10.9**	**5.0**
	[8.4,12.1]	[8.0,11.5]	[9.1,12.5]	[7.3,9.9]	[6.4,9.4]	[3.0,7.0]	[2.4,6.3]	[2.7,5.7] ^∗^	[2.1,12.6] ^∗^	[4.5,7.3]	[2.5,6.5]	[5.0,8.0]	[8.0,13.0]	[2.3,12.8]
Ma	**10.0**	**11.8**	**10.6**	**8.0**	**8.3**	**7.6**	**5.8**	**3.0**	**4.2**	**3.7** ^∗^	**2.9**	**7.5**	**3.2** ^∗^	**7.7**
	[7.8,13.7]	[10.3,13.3]	[9.4,12.0]	[7.1,9.1]	[7.3,9.6]	[6.6,8.7]	[4.5,7.3]	[2.3,7.1]	[3.1,5.6]	[2.8,6.1] ^∗^	[2.0,5.1]	[6.2,8.7]	[2.7,9.4] ^∗^	[6.3,9.0]
BH	**10.4**	**9.7**	**9.5**	**10.7**	**8.3**	**9.8**	**9.3**	7.8	**7.8**	**8.2**	**8.7**	**10.2**	**8.8**	**8.0**
	[9.3,11.8]	[8.6,11.2]	[8.5,10.8]	[9.6,12.2]	[7.5,9.4]	[8.7,11.4]	[8.3,10.5]	[6.7,9.1]	[6.7,9.0]	[7.2,9.6]	[7.7,10.1]	[8.8,12.4]	[7.8,10.0]	[6.6,9.6]
RJ	**9.1**	**7.3**	**6.5**	**8.8**	**4.9**	**8.0**	**6.0**	**4.1** ^∗^	**4.5**	**3.1**	**2.6**	**6.3**	**7.8**	**2.6** ^∗^
	[7.8,10.6]	[5.8,9.0]	[5.0,8.2]	[7.5,10.6]	[2.9,7.4]	[6.7,9.5]	[5.0,7.2]	[3.2,7.1] ^∗^	[2.8,6.5]	[2.3,6.2]	[2.2,6.0]	[5.2,7.6]	[6.4,9.2]	[2.1,9.3] ^∗^
Ca	**7.3**	**6.7**	**9.5**	**18.7** ^∗^	**2.2** ^∗^	**4.5**	**8.4**	**7.1**	**3.1** ^∗^	**9.7**	**8.2**	**9.4**	**8.5**	**9.1**
	[0.0,9.9]	[4.8,8.7]	[7.5,12.1]	[17.2,20.3] ^∗^	[1.7,20.1] ^∗^	[0.9,8.0]	[7.0,9.9]	[3.4,12.2]	[2.6,13.7] ^∗^	[8.2,11.8]	[7.0,9.8]	[8.1,11.3]	[7.4,9.9]	[8.0,10.7]
RP	**11.0**	**11.5**	**10.4**	**8.8**	**12.2**	**9.3**	**10.1**	**8.9**	**7.9**	**6.3**	**6.0**	**7.8**	**7.4**	**8.7**
	[9.7,12.7]	[10.3,12.8]	[9.2,11.9]	[7.9,10.1]	[10.8,13.7]	[8.1,10.9]	[8.7,12.0]	[7.8,10.3]	[6.7,9.1]	[5.2,7.8]	[4.9,7.5]	[6.7,9.1]	[6.1,8.8]	[7.3,10.4]
CG	**8.1**	**7.7**	**9.4**	**12.3**	**12.8**	**7.9**	**7.8**	**8.1**	**6.5**	**5.9**	**3.2** ^∗^	**8.5**	**8.4**	**4.7**
	[6.8,9.5]	[6.5,8.9]	[8.4,10.6]	[0.0,17.0]	[11.0,13.8] ^∗^	[6.6,9.2]	[6.6,9.2]	[6.0,10.2]	[4.9,8.3]	[4.8,7.3]	[2.7,7.4] ^∗^	[7.4,10.1]	[7.2,9.9]	[2.5,7.2]
Cu	**10.0**	**5.3**	**4.9**	**13.3**	**9.6**	**6.2**	**3.3**	**2.6** ^∗^	**2.4** ^∗^	**2.7** ^∗^	**2.2** ^∗^	**5.6**	**5.7**	**3.4** ^∗^
	[8.9,11.4]	[3.9,7.1]	[3.9,6.1]	[12.6,14.1]	[8.4,11.1]	[5.2,7.4]	[2.4,7.2]	[2.3, 5.5] ^∗^	[2.0,5.4] ^∗^	[2.0,5.3] ^∗^	[1.9,3.5] ^∗^	[4.6,7.0]	[4.3,7.5]	[2.8,7.7] ^∗^
Go	**9.1**	**6.4**	**8.5**	**9.1**	**8.1**	**7.0**	**8.2**	**7.3**	**8.2**	**8.0**	**6.9**	**8.0**	**8.8**	**9.1**
	[8.0,10.4]	[4.8,8.0]	[7.4,9.8]	[8.1,10.6]	[6.8,9.6]	[5.6,8.6]	[7.1,9.6]	[5.9,8.9]	[6.8,9.6]	[6.5,9.6]	[5.1,8.7]	[6.4,9.8]	[7.5,10.4]	[7.9,10.6]

The letters Fo, Na, Ma, BH, RJ, Ca, RP, CG, Cu and Go are the abbreviations for Fortaleza, Natal, Maceió, Belo Horizonte, Rio de Janeiro, Campinas, Ribeirão Preto, Campo Grande, Cuiabá, Goiânia, respectively. Non Applied (NA) means that neither the maximum value of age nor the confidence interval was found. The symbol ^∗^ highlights the values obtained after a cubic spline interpolation was done in the data. For the others values, a linear interpolation was used. Human mortality *μ*_*h*_=1/75 years ^−1^

## Results

**Fig. 1:**
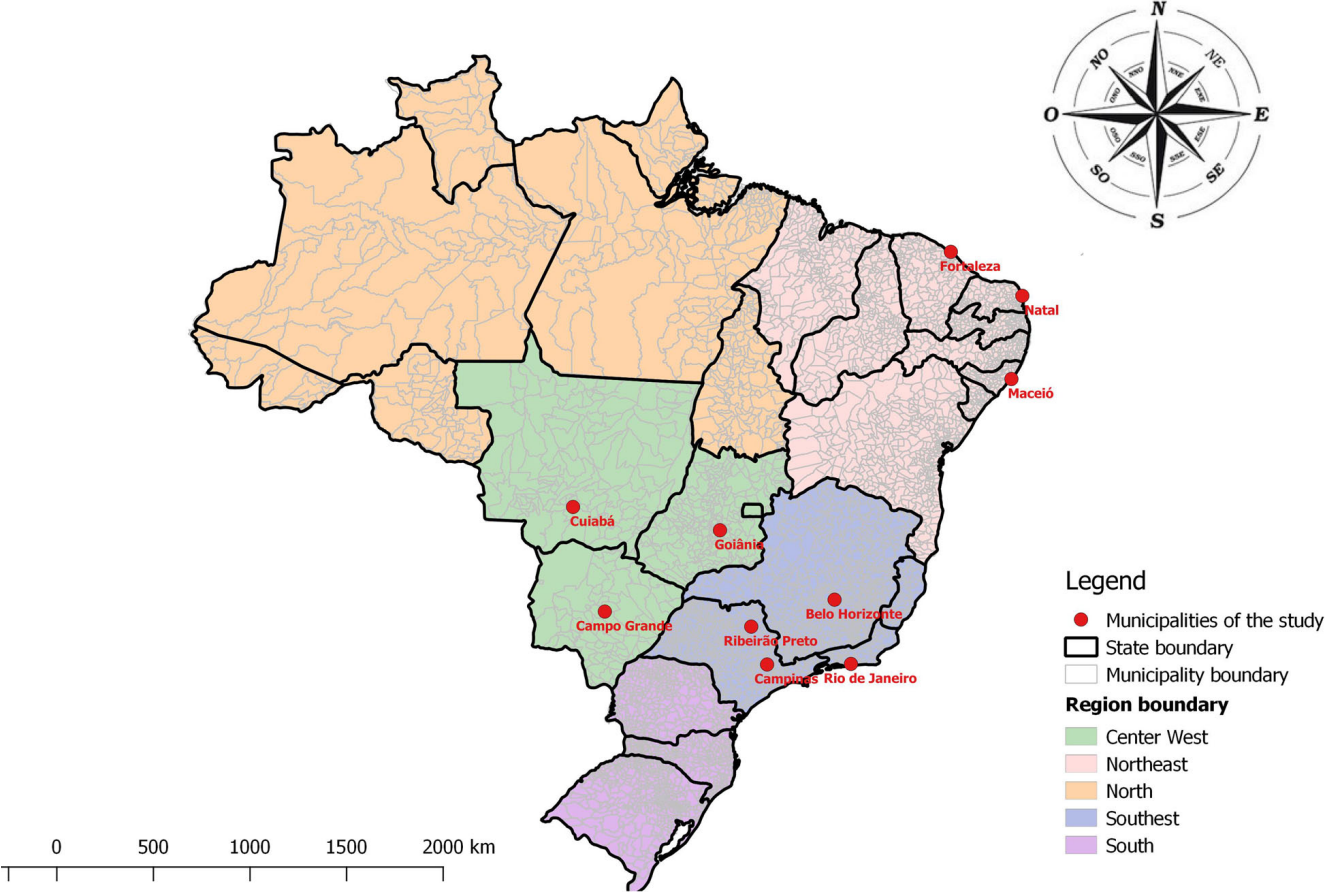
Places where data were collected. Map of Brazil with the cities studied highlighted: Rio de Janeiro (State of Rio de Janeiro), Ribeirão Preto and Campinas (State of São Paulo) and Belo Horizonte (State of Minas Gerais) belong to southwest region of Brazil; Fortaleza (State of Ceará), Natal (State of Rio Grande do Norte) and Maceió (State of Alagoas) belong to northeast region; Goiânia (State of Goiás), Cuiabá (State of Mato Grosso) and Campo Grande (State of Mato Grosso do Sul) belong to the midwest region (Source: cartographic base of Brazil in Brazilian Institute of Geography and Statistics (IBGE) [23])

Only 38 from 5565 cities, corresponding to 0.7% of the Brazilian cities, meet the minimum population criterion described in the section of Methods. Among them, 10 from the 38 cities are selected since they also follow the criterion of having cumulative incidence above or equal to 500 per 100,000 habitants during the studied period. The chosen cities belong to different states and they are localized in three of the five geographic regions of Brazil (see Fig. 1): Rio de Janeiro (RJ), Ribeirão Preto (RP), Campinas (Ca) and Belo Horizonte (BH) belong to the Southwest Region; Fortaleza (Fo), Natal (Na) and Maceió (Ma) are localized in the Northeast Region; Goiânia (Go), Cuiabá (Cu) and Campo Grande (CG) are from the Midwest Region. These cities are responsible for 22.8% (1,779,350/7,818,624) of dengue cases reported over the 14 years period.

**Fig. 2:**
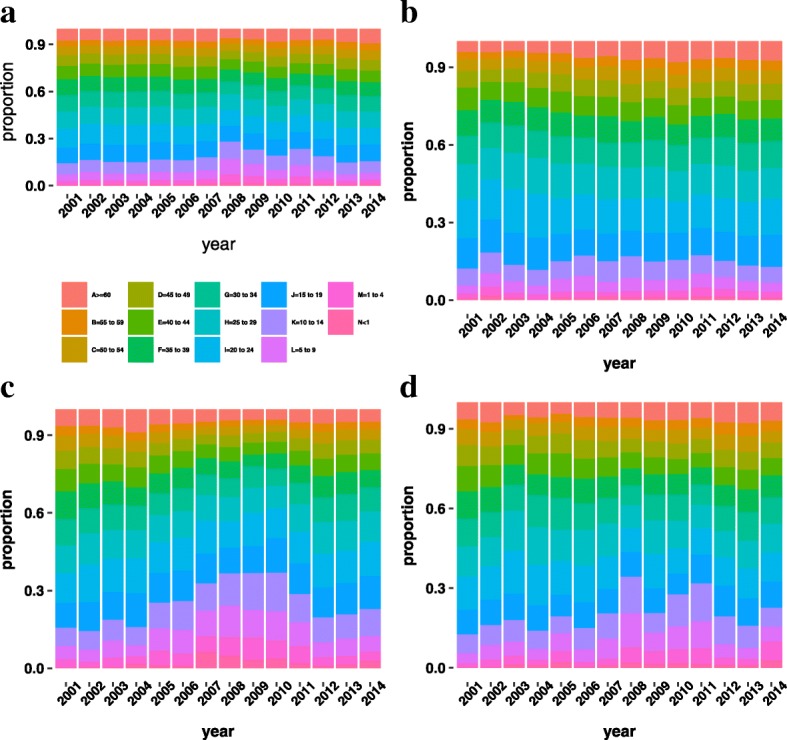
Distribution of dengue cases by age and time. Age distribution of Dengue cases from 2001 to 2014 for: **a**) the whole country; **b**) Goiânia (in the Midwest Region); **c**) Fortaleza (in the Northeast Region); **d**) Rio de Janeiro (in the Southeast Region)

Figure 2 illustrates the age distribution of dengue cases in the whole country and in three other studied cities selected from each region of the country. Distinct patterns can be observed among the cities and for the same city in different years. In particular, we can see that the age profile for the proportion of dengue cases in Goiânia (Fig. 2b) is homogeneous during the 14 years period. Also, 25% of the cases occur in individuals that are under 19 years old. For Fortaleza (Fig. 2c), we can see that between 2005 and 2010 there was a substantial increase of the proportion of dengue cases over small age classes (until 14 years old). Among the studied cities, Rio de Janeiro (Fig. 2d) had the most heterogeneous age profile for the proportions of dengue cases.

**Fig. 3:**
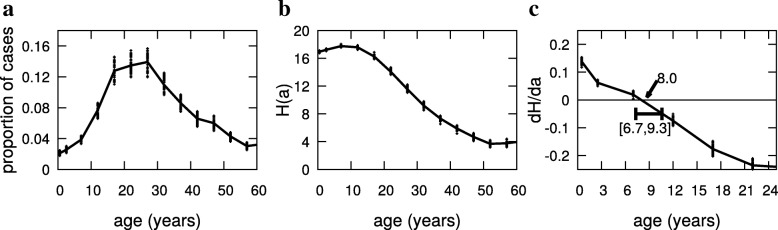
Age distribution of cases, and evaluation of the optimum age for Dengue vaccination. An example of the applied procedure of estimation of optimal age and its confidence interval: **a**) the proportion of dengue cases by age *a*; **b**) *H*(*a*) as a function of age *a*; **c**) the derivative of *H*(*a*) with respect to age *a*. The data correspond to dengue cases in Maceio in 2014. The dotted line connecting the data is a linear interpolation, and the symbol (+) corresponds to simulated curves. The optimal age is 8.0 years, and the confidence interval is [6.7,9.3]

In order to illustrate the procedure used to obtain the optimal vaccination age and its confidence interval we show in Fig. 3 the proportion of dengue cases by age, the curve *H*(*a*) as a function of age, and the derivative of the curve *H*(*a*) with respect to age, *h*(*a*). The three curves correspond to the proportion of dengue cases in Maceió reported in 2014. For this data set, we estimated the value of the optimal vaccination age as 8.0 years (Fig. 3b) and from 6.7 to 9.3 its confidence age range (Fig. 3c).

Table 1 summarizes the results obtained for the optimal age and its confidence age range across the cities and years assuming *μ*_*h*_=1/70.5 years ^−1^. We also present the result for the whole country. The confidence age range varies from less than 1 year (Campo Grande and Cuiabá, 2004, and Fortaleza, from 2007 to 2011) to 20 years in Campinas (2005). The optimal vaccination age varies from 2 years old to 19 years old in Campinas (2004). Concerning the whole country, the minimum and maximal values for the confidence intervals should be from 2 years old (2008) to 10 years old (2001, 2004, 2013-2014), meanwhile the optimal age is from 3 years old (2008) to 8 years old (2001, 2003-2004, 2013-2014).

Table 2 shows the confidence age range of the optimal vaccination age across the cities and years assuming the other extreme value (1/75.0 years ^−1^) of the human mortality rate *μ*_*h*_. The confidence age range varies from less than 1 year old (Campo Grande, 2004, Fortaleza, from 2007 to 2011, and Campinas, 2001) to 20 years old in Campinas (2005). The optimal vaccination age varies from 2 years old to 18 years old in Campinas (2004). Concerning the whole country, the only difference regards the maximal value of the confidence interval that decrease from 10 (<10.5) years old in Table 1 to 9 (> 9.5) years old in Table 2 (2001, 2004, 2013-2014).

**Fig. 4:**
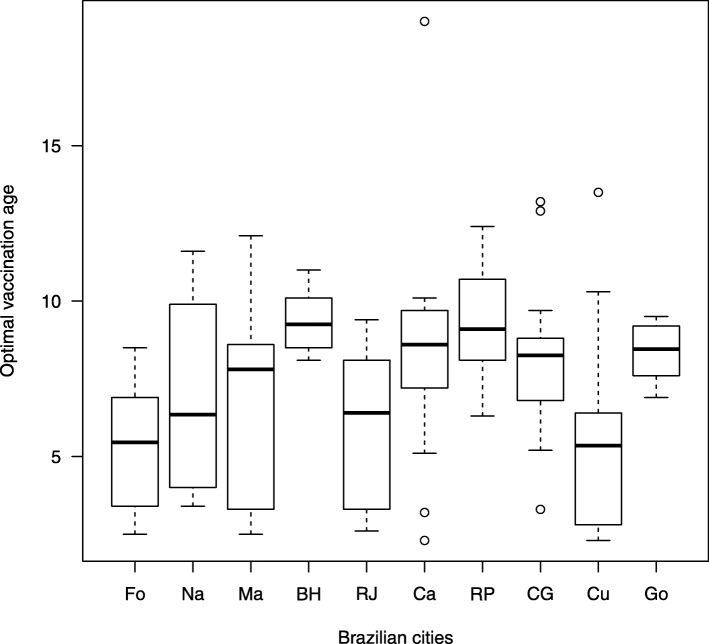
Box plot of the optimal age for Dengue vaccination. Box plot of the optimal age for the studied cities from 2001 to 2014; the letters Fo, Na, Ma, BH, RJ, Ca, RP, CG, Cu and Go are the abbreviations for Fortaleza, Natal, Maceió, Belo Horizonte, Rio de Janeiro, Campinas, Ribeirão Preto, Campo Grande, Cuiabá, Goiânia, respectively. The bold lines correspond to the median values of the optimal vaccination age shown in Table 1. The boxes represent the dispersion and the skewness of the optimal ages along the studied period except the outliers, which are marked by circles. The intervals between minimum and maximum values of the optimal ages are determined by the dashed lines ends

Figure 4 shows the behaviour of the optimal vaccination age for the ten Brazilian cities during the study period (2001 to 2014) through a box plot representation of the optimal age exhibited in Table 1. Although the results exhibited in Table 2 are slightly different for optimal age and confidence interval, the variation from the minimal to the maximal value of *μ*_*h*_ does not produce significant changes in Fig. 4, which compiles, after all, the optimal age and its dispersion and skewness for each city taking into account the studied period. Concerning the different scenarios of the studied cities, Fig. 4 also shows that the median value of the optimal vaccination age is around 9 years old in Belo Horizonte, Campinas, Ribeirão Preto, Campo Grande and Goiânia; moreover their dispersion and skeewess are smaller than for the rest of the studied cities. On the other hand, the median value of the optimal vaccination age is around 6 years old in Fortaleza, Natal, Rio de Janeiro and Cuiabá; the largest dispersion is observed in Natal, meanwhile Maceió presents the largest skeewess.

## Discussion

In this work, a partial differential equation model with age-structure was used to evaluate the optimal vaccination age in ten selected Brazilian cities as well as in the whole country. It is worth to note that, currently, an efficient vaccine with no restriction does not exist. The vaccine licensed in some countries, including Brazil, is indicated to aged from 9 to 45 years old, and recommended only to individuals with previous dengue infection, due to the highest risk of hospitalizations in dengue naive individuals [19, 20].

Considering scenarios where everybody is vaccinated at the same age, vaccine efficacy is constant along the age classes, and the same for all serotypes; the priority age range of vaccination against dengue, in the 10 Brazilian cities of this study, varies from less than one year old to 20 years old. The optimal vaccination age indicates that vaccination has to target individuals under 19 years old according to the results presented in Table 1 (that suffered a slightly reduction to 18.7 years old in Table 2). If the outliers are not considered, according to Fig. 4, vaccination has to target individuals under 13 years old; therefore we have assumed age of 13 as the maximum age for dengue vaccination.

As far as we know, this is the first time that a mathematical model, based on PDE, is applied to dengue epidemics using actual age distribution of cases in different cities, promoting a general and comparative scenario of many epidemics in those Brazilian cities during a time interval of more than 10 years (from 2001 to 2014). The variation of the optimal age for vaccination across the country highlights the complex spatial-temporal behaviour of the disease, which is evident in the results illustrated in Fig. 4 as well as in Table 1. For instance, in the case of Fortaleza, comparing Fig. 2c to results presented in Table 1 and Fig. 4, the decreasing of optimal age seems to be related to the increasing of children infected by dengue, which is also observed in other cities of the Northeast of Brazil [21].

The results are compatible with others from the literature. For instance, the authors in reference [9] applied an age dependent seroprevalence model to dengue data of a Northeast Brazil city, founding a similar age range for vaccination (3 to 14 years old). Due to lack of age distribution of dengue cases by serotype for each studied city, our model does not consider the four serotypes. However, our results are also compatible with others from the literature that considers co-circulation of dengue serotypes, since the authors obtain that the target age range is from under 1 year to 9 years old, for the whole country [13]. That comparison was allowed due to the values of optimal age (from 3 to 8 years old) and confidence interval (from 2 to 10 years old) estimated for the whole country in Table 1. Although that result for the whole country should not be applied for all cities with dengue cases, it could be a coarse approximation for other cities (besides the ten studied cities) that have large population and large incidence. It is a kind of ’spatial’ mean field result to provide a general view of the country with the aim of comparing with the results in the literature.

The model, as well as the method of estimating an optimal age, was previously applied to dengue data in Mexico (the whole country) in 2014 using an analytical fitted function for the age distribution [14]. Since the PDE model allows the variables to change continuously with the age, it is possible to calculate the age interval for priority of dengue vaccination for each city. In this work we take advantage of the numerical approach to evaluate the proportion of dengue cases by age instead of founding an analytical function. As Cruz-Pacheco et al. [14] we find out another important point that should be emphasized: the optimal age for vaccination does not match with the age of highest number of cases, showing up that this quantity is not a good criterion to prioritize the population to be firstly vaccinated.

Since the results are based on dengue reported cases in 10 Brazilian cities from 2001 to 2014, from the theoretical point of view, we could say that if a safe and effective vaccine should be applied before 2001 to the target population, it would have been possible to avoid the cases of dengue in children under the age of 13 (taking out the outliers above the maximal optimal age among the 10 cities in Fig. 4), as well as to reduce the incidence of dengue in adults, although this is only a plausible hypothesis. In addition, as the contingent of children under the age of 13 in Brazil is very large (around 42 millions), this would require great operational efforts from the National Immunization Program, as well as great financial resources.

Considering the whole country, these results indicate that the confidence age range of vaccination against dengue is from 2 to 10 years old, which implies that the target population is reduced to 27 millions of individuals which is still a large population for a vaccination campaign, mainly if it is needed more than one dose. Nowadays, it is not so far to dispose of a safe and effective vaccine [5, 6] and when available, possibly, there will be no scale production able to satisfy a demand of such magnitude. Moreover, it should be noted that more than a hundred countries have been affected by dengue epidemics [22], and most of them will also demand for vaccine. For these reasons, it is necessary to adapt vaccination strategies to the cost and quantity of the vaccine eventually available. Due to the limitations explained above, in the particular case of Brazil, a country with continental dimension with more than 200 millions of habitants, an alternative to reduce the target population should be to apply vaccine in areas that had already large epidemics as well as areas with higher risk of severe forms of the disease, because large proportions of the population already have antibodies against one or more serotypes of the dengue virus [7].

The method presented in this work provides another alternative using dengue notification data in different cities that meet the epidemiological diversity of dengue. For instance, using data of dengue epidemics during 2014, the confidence age range for vaccination should be from 2 to 10 years old in Rio de Janeiro, meanwhile in Fortaleza it is from 3 to 7 years old, that would correspond to fewer vaccine doses (see Table 1). Moreover, based on the statistical analysis, it is possible to consider the past history of the disease for each city in order to estimate the optimal age of vaccination and its variation along a time interval (see Fig. 4), setting up the priority age vaccination for each city based on the dispersion of the optimal age for the previous long-term time series of dengue cases. These results reveal heterogeneous scenarios of priority age vaccination for the analysed cities in a large country as Brazil. Finally, it is worth to notice that the decision should be subordinated not only to the age range defined by the model, but also to the age health restrictions of the vaccine.

The main advantage of using mathematical models to define the target population for vaccination is the possibility of addressing and checking different scenarios that may provide valuable information for the Health Public System. In addition, due to patterns variability of the age distribution of cases, the numerical approach is more suitable to be used with epidemiological data frequently updated. Finally it worth to say that it is a powerful method to consider more complex situations such as different vaccination schedules, taking advantage of the variation of parameters and functions with age.

## Conclusion

In conclusion, the findings of this study indicate that the model and methods used here, coupling mathematical methods with actual epidemics data, may constitute a valuable tool to define the age range to be prioritized for dengue vaccination, attending to the necessity of the application of minimum doses of vaccine necessary to obtain herd immunity capable of reducing transmission of the dengue virus, and consequently to control epidemics.

## Abbreviations

BH: Belo Horizonte
Ca: Campinas
CG: Campo Grande
Cu: Cuiabá
DHF: Dengue hemorrhagic fever
Fo: Fortaleza
Go: Goiânia
IBGE: Brazilian institute of geography and statistics
Ma: Maceió
Na: Natal
ODE: Ordinary differential equations
PDE: Partial differential equations
RJ: Rio de Janeiro
RP: Ribeirão Preto
SI: Susceptible, infected
SINAN: Brazilian Notified Disease Information System
SIR: Susceptible, infected, recovered
